# The Cannabis Terpenes

**DOI:** 10.3390/molecules25245792

**Published:** 2020-12-08

**Authors:** Sarana Rose Sommano, Chuda Chittasupho, Warintorn Ruksiriwanich, Pensak Jantrawut

**Affiliations:** 1 Plant Bioactive Compound Laboratory, Faculty of Agriculture, Chiang Mai University, Chiang Mai 50100, Thailand; 2 Cluster of Agro Bio-Circular-Green Industry (Agro BCG), Chiang Mai University, Chiang Mai 50100, Thailand; 3 Cluster of Research and Development of Pharmaceutical and Natural Products Innovation for Human or Animal, Chiang Mai University, Chiang Mai 50200, Thailand; chuda.c@cmu.ac.th (C.C.); warintorn.ruksiri@cmu.ac.th (W.R.); pensak.j@cmu.ac.th (P.J.); 4 Department of Pharmaceutical Sciences, Faculty of Pharmacy, Chiang Mai University, Chiang Mai 50200, Thailand

**Keywords:** essential oil, hemp, marijuana, trichomes, volatile profile

## Abstract

Terpenes are the primary constituents of essential oils and are responsible for the aroma characteristics of cannabis. Together with the cannabinoids, terpenes illustrate synergic and/or entourage effect and their interactions have only been speculated in for the last few decades. Hundreds of terpenes are identified that allude to cannabis sensory attributes, contributing largely to the consumer’s experiences and market price. They also enhance many therapeutic benefits, especially as aromatherapy. To shed light on the importance of terpenes in the cannabis industry, the purpose of this review is to morphologically describe sources of cannabis terpenes and to explain the biosynthesis and diversity of terpene profiles in different cannabis chemovars.

## 1. Introduction

*Cannabis sativa* L. or cannabis is a herbaceous annual that has a long history of use around the world as fiber, food, oil as well as medicine. Depending on the purposes of utilization, they can be called by different names; for example, “hemp” as a fiber and textile crop and “recreational cannabis”, or known in the USA as marijuana. Aside from its quality as an industrial textile, the psychoactive properties have labeled a grey stigma to cannabis plants as being an illicit drug with many informal names including pot, dope, grass, weed, Mary Jane, bud, hash, bhang, kef, ganja, locoweed, reefer, doob, spliff, toke, and roach. It has been forbidden to grow in many countries due to the psychoactive ingredients contained [1]. In a medical sense, many recent studies have advised that the increase in cannabis use was in association with psychiatric symptoms including depression and anxiety [2,3]. However, many users still exclusively endorse its recreational purpose [4,5]. As a result, there has been a strong movement toward correcting negative attitudes of the cannabis and attempts have been made in trying to remove this plant from narcotic lists. In Thailand, for instance, as from 2020, cannabis strains such as กัญชง (hemp) and กัญชา (marijuana) are legally grown for industrial fiber or medicinal purposes under the controlled level of active metabolites including cannabinoids of Δ9–tetrahydrocannabinol (THC) and cannabidiol (CBD) [6]. While focus has paid attention primarily to the bioactive functions of the cannabinoids, the hydrocarbon terpenes could also offer interesting entourage effects that could ideally synergize or downstream their effects [7]. Eminently, with the rise in the legal cannabis industry, interest has been made around cannabis terpenes as they contribute many of the different aromatic characteristics that influence the diverse varieties of cannabis strains [8]. Within the scope of this review, we provide the general background history of cannabis discovery and the importance of the terpenes. The taxonomy and morphology of the cannabis, particularly the localization of the terpenes, are discussed. More importantly, the chemistry, biosynthesis, and diversity of terpenes in different cannabis genotypes are of major interest in this review. 

## 2. The Cannabis Discovery and Its Importance as a Source of Terpene

**Figure 1:**
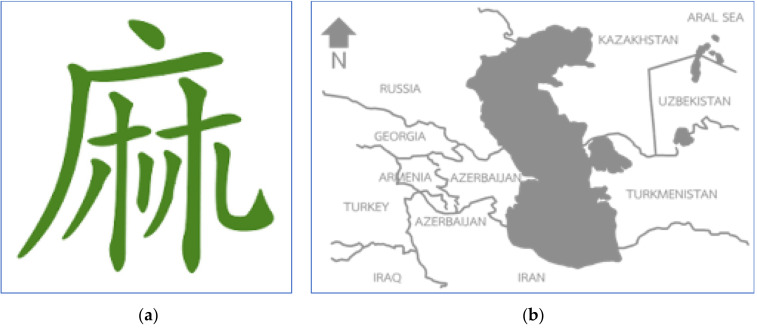
The evolution of the Chinese characters of the word “*Cannabis sativa*” or “Ma” (**a**) and the place of origin of the cannabis plant in the southern Caspian sea near Iran (**b**).

Cannabis has a long history dating back approximately to just after the Ice Age as cord and textile scraps made of cannabis fiber have been found in historic caves in the Czech Republic [9,10]. In the 19th century, the plant was recorded as originating near the southern area of the Caspian Sea near Iran (Figure 1b) [11,12]. It was later confirmed by the chemotaxonomy of the essential oil from cannabis of diverse origins and most of the cannabis phenotypes collected around the globe had chemical ingredients similar to those of Central Asian origin [13]. In previous days, it was known as the original fiber plant in Asian culture. Seed and seed oil extracts were also used as food [11,14]. The first record of cannabis in the literature in China can be dated to approximately 5000 years ago as written by emperor Chen Nong who was then known as the father of Chinese agriculture. The Chinese alphabet “Ma” was created using the mimic of the cannabis drying process (Figure 1a). The letter was adjusted to describe the male plant, “his” separated from the female plant, “chu” for the quality of the fiber [15,16]. In 500 BC, the use of cannabis spread worldwide from Asia to Europe and to Africa through the silk road. In the 19th century, hemp was popular in the western world as a fiber crop that had superior qualities [17,18]. It is not too surprising therefore that cannabis is today known as an ideal plant in terms of sustainability. In a period of rapid industrial growth, hemp became the industrial crop as countries raced against one another toward modernity. In addition to the world textile industry, the plant began to be known for medical issues. It was believed that during the 19th century, there were thousands of cannabis medicines available, produced by more than 280 manufacturers [19]. The growth and the interest in this fiber crop crashed after the attempt to add it to the narcotic list with opium during the Geneva convention in 1925 [19]. 

The first cannabinoid isolates used for medicinal purposes was in Czechoslovakia, and CBD was fully characterized for the first time in 1963, followed by the psychoactive THC in the following year [20,21,22]. The discovery of cannabinoid receptors, CB1 and CB2 together with the full comprehension of the endocannabinoid system helped us recognize the medicinal benefits of this plant [23,24]. In 1942, Simonsen and Todd [25] were the first researchers who put terpene fractions as a separate category from the cannabinoids and *p*-cymene was reported as a main constituent from Egyptian hashish. Only in the past years have the terms synergic and/or entourage effect of the other cannabis compounds including those of the terpenes been speculated by chemists all over the world [26]. It was first described as routes for the molecular regulation of endogenous cannabinoid activity [27]. Russo [28] documented the unique therapeutic effect of cannabis terpenes that possibly played a role on the entourage effects of the medicinal properties of the cannabinoids. This phytocannabinoid-terpenoid synergy could enhance the treatments of pain, inflammation, depression, anxiety, addiction, epilepsy, cancer, fungal, and bacterial infections [7,28,29,30,31]. 

## 3. Taxonomy and Localization of the Cannabis Terpenes

**Figure 2:**
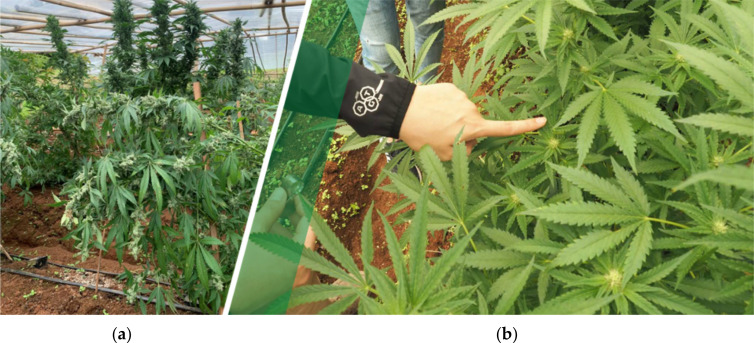
The cannabis plant var. Kees’ Old School Haze^®^ (available at https://dutch-passion.com) (**a**) and their flower buds var. Gorilla Glue (available at http://www.seedstockers.com) (**b**).

Cannabis belongs to the small family of Cannabaceae, which includes hop and Celtis berry. It is a dicotylate angiosperm that gives incomplete (lacking of petals) and also imperfect flower types with the separated sexual organs. The flower bears only pistils, known as pistillate flowers (or female flowers), and those with only the stamens are called staminate (or male flowers). In nature, cannabis produces either male or female flowers (dioecious), however, under short-day conditions, it may give both male and female flowers or monoecious. Plants are able to grow as high as 3 m or smaller depending on the varieties and the conditions of growth. The flowers often initiate as groups of flowers or as an axillary bud (Figure 2 a,b). The stem consists of the outermost layer of the epidermis, which is thin and coarse, and the primary and secondary layers that provide the better fiber quality. The innermost, which is the lignified secondary blast fiber, give the best fiber quality [32,33]. 

**Figure 3:**
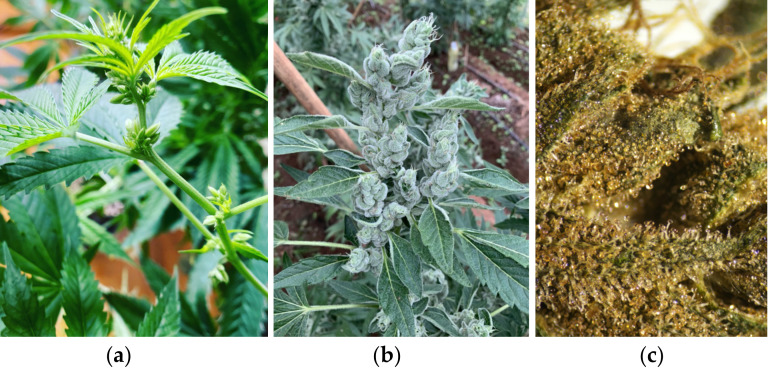
The cannabis staminate flowers (**a**) and pistillate flowers (**b**) with the trichome structures as the localization of cannabinoids and terpenes of Kees’ Old School Haze^®^ (available at https://dutch-passion.com) (**c**).

Leaves are palmate with 5–7 leaflets. The male flower (Figure 3a) has no petals, usually with five yellowish tepals, and five anthers yielded pollen. The female flower (Figure 3b) had a single-ovulate and was enriched with the trichome structures, which are the localization of the cannabinoids and the terpenes as shown in Figure 3c. These terpenes are responsible for the defense and interaction with herbivores and pests.

Taxonomists classified cannabis plants into three species in the early days: (i) *C. sativa* Linnaeus, (ii) *C. indica* Lamarck, and (iii) *C. ruderalis* [34,35]. Today, many researchers are convinced that the cannabis that grows commercially is *C. sativa* L, but the subvariety “sativa” should be known as hemp and the subvariety “Indica” should be called recreational cannabis or marijuana. The differences in these subvarieties are shown in Table 1. The usable part for hemp is the stem in particular, while parts usually with trichomes are the usable parts for the cannabis. The level of THC is graded as >2% of dry weight and flowers give a much higher terpene content, which becomes sticky to the touch [36]. Some researchers do not agree with the separation of the two by the chemical compositions. Morphologically, the leaf of cannabis is broader and the color is darker compared to that of hemp. Many recent studies have attempted to separate the combination of the terpene composition in several species of Cannabaceae where it is apparent that hemp and the Indica cannabis are closely related. For example, hemp can also yield terpene profiles similar to those of marijuana [37]. 

**Figure 4:**
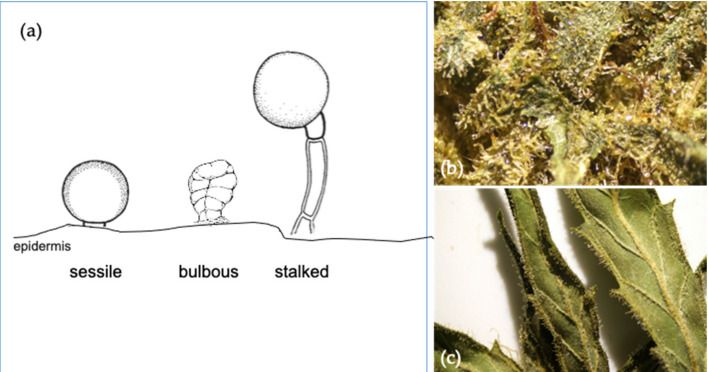
Different trichome structures for cannabis plants (**a**); the stalked types that are available on the floral surface (**b**); and sugar leaf structures with the presences of trichomes of the *Cannabis sativa* L. var. Kees’ Old School Haze^®^ (available at https://dutch-passion.com) (**c**).

Three types of glandular trichomes are characterized based upon their surface morphology, namely bulbous, sessile, and stalked (Figure 4a) [38]. Bulbous trichomes are the smallest, while sessile trichomes appear on the epidermis with a short stalk and globose head comprised of a multicellular disc of secretory cells and a subcuticular metabolite storage cavity. Similarly, the stalked trichomes are slightly larger with a globose head elevated above the epidermal surface by a multicellular stalk [39]. In cannabis, the sessile and stalked trichomes differ not only in morphology, but they also have distinct fluorescent properties, number of cells in their secretory disc, and terpene profiles [40]. The stalked glandular trichomes of mature flowers have a globular head consisting of an enlarged disc greater than eight secretory cells known to be rich in cannabinoids and monoterpenes (Figure 4b). The sessile trichomes are mainly found on sugar leaves (Figure 4c). They have eight secretory cells that produce less cannabinoids and higher proportions of sesquiterpenes.

To separate the trichomes, the flowers are usually pre-frozen or freeze-dried and then are gently rubbed on the sieve mesh. The trichomes separated in this process are known as kief, which can be pressed to make hash. In Nepal, hash is hand-shaped into balls, also known as wax or “Charas” [41]. Hash oil, on the other hand, is the concentrated hash that has been dissolved in organic solvents such as alcohol, propane, or butane [42,43]. The extraction allows pigments such as chlorophylls and other contaminants to be extracted along with the terpenes, resulting in a dark green color extract. After extraction, the solvent is then removed by evaporation either by direct heat or under a vacuum, resulting in the oil product with high viscosity. 

## 4. Terpene Biosynthesis in Cannabis

**Figure 5:**
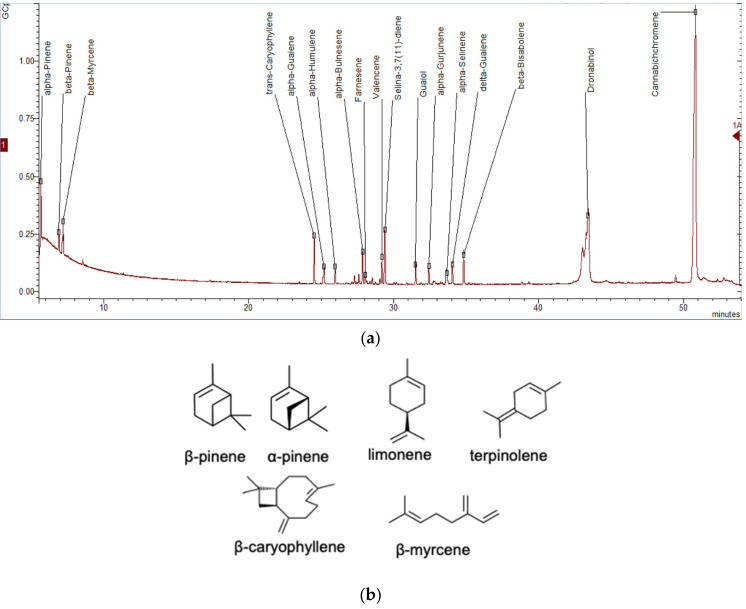
The gas chromatogram equipped with mass spectrometry (GC-MS) of the cannabis terpene extract (butanol) from the floral tissue of *Cannabis sativa* L. (**a**) and predominant terpene chemovars (**b**). Denotes the dried cannabis flower (0.2 g) extracted with propanol by the ultrasonic assisted method [36] and gas chromatography mass spectrometry (GC-MS) analysis was performed using the protocol described previously [57].

The energy required for plant growth and development derives from photosynthesis, respiration, and transpiration with O_2_, CO_2_, nutrients, and water. The energy is restored in the form of primary chemical ingredients that plants later exploit. These primary metabolites include carbohydrates, lipids, proteins, and nucleic acids. However, during cycles of growth and reproduction, plants might be challenged by stresses including hard environmental conditions or pests and herbivores. Plants then produce different groups of compounds called secondary metabolites that are used as defenses to those challenges. For example, it can produce compounds that draw in pollinators including birds to help them in the fertilization process or seed dispersion [44]. These compounds are produced in different forms and are exploited for their biological functionalities [45]; for example, alkaloids such as morphine and codeine in opium give psychoactive and pain relief activity to mammals. Phenolics and flavonoids found in the skins of fruits and berries possess antioxidant activity [46]. Sulfur containing compounds such as allicin in garlic can be used to reduce lipoglycerides in the blood and also have the ability to stimulate appetite [47]. Saponin glycoside in soap nuts can be used as a surfactant [48], and finally, the terpenoids, which are main ingredients found in plants containing essential oils [49], are used as food additives and some depict psychoactive ability and aroma characteristics such as those found in the cannabis. Terpenes are hydrocarbons with small isoprene units linked to one another to form chains, while terpenoids are oxygen-containing terpenes. Three types of terpenes/terpenoids are usually found in the cannabis plant which are (i) monoterpenes (10C) of two isoprene units; (ii) sesquiterpenes (15C) of three isoprenes; (iii) diterpenes (20C) of four isoprenes; and (iv) triterpenes (30C) of six isoprenes [26]. To date, more than 200 volatiles have been reported from the different cannabis genotypes of which 58 monoterpenes and 38 sesquiterpenes have been characterized [50,51,52,53]. Figure 5a illustrates a chromatogram of the terpene extract from the floral tissue of cannabis. Among others, the major monoterpene components are limonene, β-myrcene, α-pinene, and linalool with traces of α-terpinolene and tran-ocimene [54,55] (Figure 5b), while predominate sesquiterpenes are *E-*caryophyllene, caryophyllene oxide, *E*-β-farnesene, and β-caryophyllene [56]. The cannabinoids are biologically synthesized from diterpene structures to form phenol terpenoids, which account for almost a quarter of all metabolites [26]. Thus, the combination of the terpenes provides the unique aromas to different strains.

**Figure 6:**
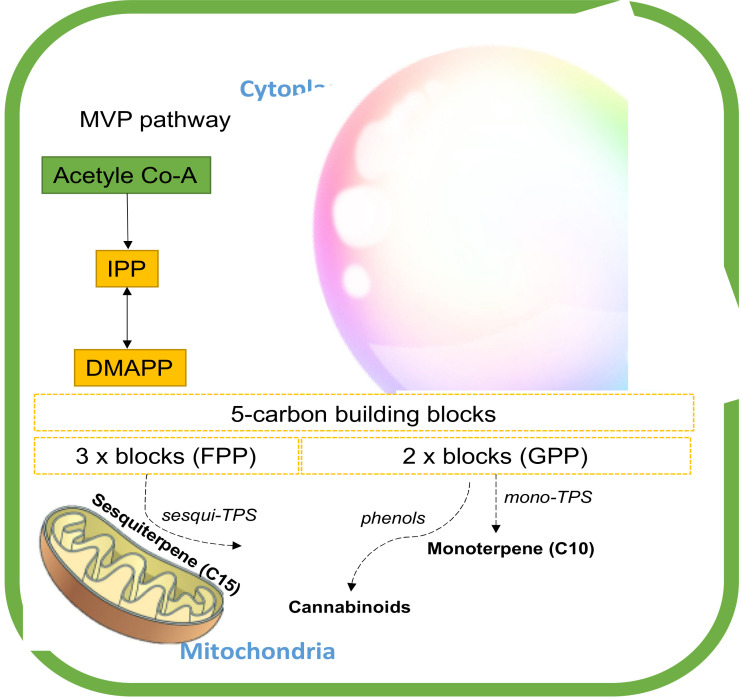
The terpene biosynthesis pathway in cannabis. Abbreviations: DMAPP, dimethylallyl diphosphate; FPP, farnesyl diphosphate; IPP, isopentenyl diphosphate; GPP, geranyl diphosphate; mono-TPS, monoterpene synthase; sesqui-TPS, sesqiterpene synthase.

The biosynthesis of these secondary-metabolite terpenes starts with the common isoprenoid diphosphate precursors (5C) through two paths, the plastidial methylerythritol phosphate (MEP) pathway and the cytosolic mevalonate (MEV) pathway [8,58]. These pathways regulate the different substrates available for terpene synthesis (TPS). The MEP converts pyruvate and glyceraldehyde-3-phosphate (G3P) into 5-carbon building blocks, isopentenyl diphosphate (IPP), and dimethylallyl diphosphate (DMAPP) in the plastids [59]. The MEV pathway, on the other hand, alters three units of acetyl-CoA to IPP, which is then isomerized to DMAPP by IPP isomerase in cytosol. IPP and DMAPP are condensed into longer-chain isoprenoid diphosphates that include geranyl diphosphate (GPP) and farnesyl diphosphate (FPP) [58]. These linear isoprenoid diphosphates are substrates for monoterpene synthases (mono-TPS) and sesquiterpene synthases (sesqui-TPS), respectively, which diversify these precursors through enzymatic modifications such as hydroxylation, dehydrogenation, acylation, and glycosylation into the diverse ranges of mono- and sesquiterpenes [59,60]. GPP is also a building block of cannabinoid biosynthesis (Figure 6) [61].

The biosynthetic pathway of cannabinoids involves the chemical joining process of the phenol with the terpenes to form the non-activate acidic forms that largely determine their potency and pharmaceutical properties including cannabichromene (CBC), cannabidiolic acid (CBDA), cannabigerol (CBG) cannabinol (CBN), cannabidivarin (CBDV), cannabidivarinic acid (CBDVA), cannabigerolic acid (CBGA), cannabicyclol (CBL), delta 8*-*THC, tetrahydrocannabinolic acid (THCA), and tetrahydrocannabivarin (THCV) [62]. These compounds, along with the terpenes, are produced in the trichome structures available on the female cannabis flower [40]. The highest concentration of the natural cannabinoids in cannabis are cannabidiolic acid (CBDA) and Δ9-tetra-hydrocannabinoic acid (Δ9-THCA). The psychoactive metabolites such as delta 9-THC and the non-psychoactive CBD are then activated through decarboxylation by heat treatments. It is also favored by several factors such as storage time and the use of alkaline conditions [63,64]. Below are the important cannabis terpene groups and their synergistic and functional properties.

### 4.1. Cannabis Monoterpene

The α-pinene and β-pinene inhibits the activity of acetylcholinesterase in the brain. Therefore, it is claimed to aid memory and minimize cognitive dysfunction induced by THC intoxication [65]. The characteristic of pine scent possesses antiseptic activity [49,66,67]. β-myrcene is known to have the analgesic effect of THC and CBD by stimulating the release of endogenous opioids through the α2-adrenergic receptor dependent mechanism [68,69]. Thus, if the level of myrcene is >0.5%, it may result in a “couch lock” effect while low levels of myrcene (>0.5% myrcene) can produce a higher energy [26]. This compound offers the musky or hop-like fragrance with the functions of antioxidant and anticarcinogens [28,66]. Even though it has been postulated that limonene of the citrus aroma has a low affinity for cannabinoid receptors, this monoterpene boosts up the level of serotonin and dopamine, thereby inducing the anxiolytic, anti-stress, and sedative effects of the CBD [68,70]. The floral fragrance of linalool could assist with the anxiety through aromatherapy [66].

### 4.2. Cannabis Sesquiterpenes

β-Caryophyllene, a spice (pepper) aroma, is the most available sesquiterpenoid in cannabis plants and extracts, especially after decarboxylation by heat. It is an agonist with the CB2 receptor without psychoactivity [52]. It is also responsible for the cannabis anti-inflammatory effects [66]. This sesquiterpene is also proven to give gastroprotective, analgesic, anticancerogenic, antifungal, antibacterial, antidepressant, anti-inflammatory, antiproliferative, antioxidant, anxiolytic, analgesic, and neuroprotective effects [26]. The caryophyllene oxide that gives the lemon balm-like scent is proven to have anti-fungal and insecticidal properties [28]. 

## 5. The Cannabis Chemovars

**Figure 7:**
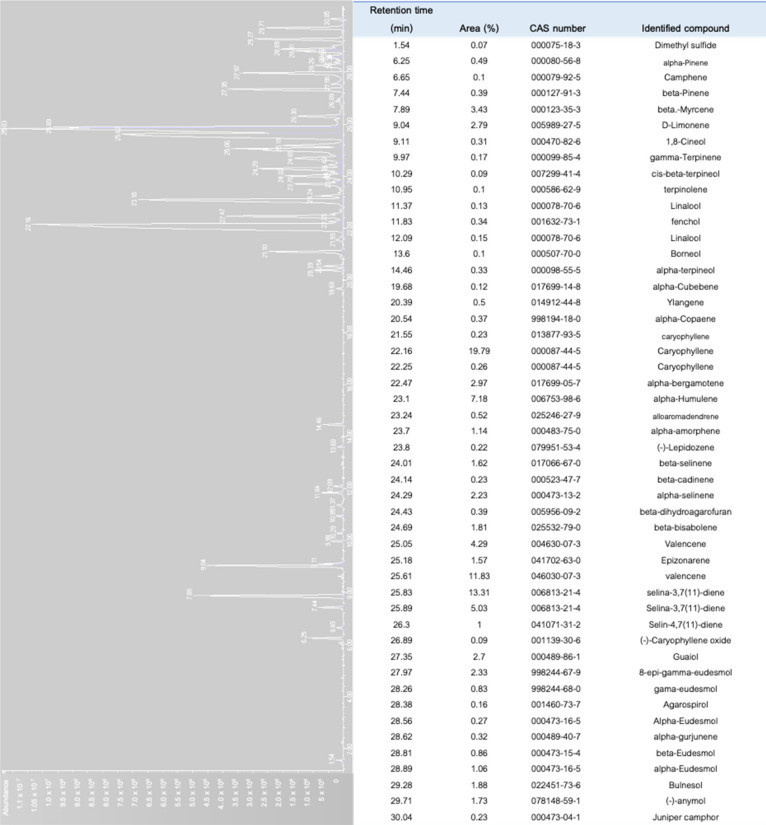
The chromatogram of the volatile profiles of *Cannabis sativar* L. var. Northern Light (https://www.seedstockers.com). Denotes terpenes isolated from dried florescence (50 mg) using headspace solid phase microextraction (SPME) with 50/30 μm carbowax-divinylbenzene, CAR-PDMS-DVB StableFlex fiber (Supelco, Bellefonte, PA, USA) followed by gas chromatography coupled with mass spectrometry [75,78].

Depending on the variable compositions of the terpenes, different cannabis ‘strains’ elicit different aromas with a greater link to product quality, retail price, and consumer preference [8,71]. The terpene compositions of cannabis are a seasonal variable. The alteration in the proportion of terpenoids in cannabis are in accordance with the variety of cannabis, plant part, environmental conditions, maturity, and method of analyses [72,73,74]. Different growth stages of the cannabis could give considerable variations in the terpene compositions. The terpene profile of the cannabis at the vegetative stage was considered to have a much lower proportion of monoterpenes than the flowering stage [56]. Aside from the variations and compositions of terpenes among different phenotypes, the modulated molecular or biological functions of the terpenes are effective only when the concentration of the terpene in the full-spectrum cannabis extract is above 0.05% v/w [31,68,75]. To characterize the aromatic profile of the cannabis of different chemovars, solid phase microextraction (SPME), which is non-destructive and non-invasive, was used to collect the volatiles from the samples [53,76]. This method favors a small sample size, and eliminates the use of organic solvents and more importantly, it allows for the emission of hundreds of volatile compounds from the samples [53,77]. Figure 7 shows the chromatograms of the volatile compounds diffusing from the floral tissue of *C. sativa* var. Northern Light using SPME and a gas chromatography. As many as 51 volatiles were detected in which caryophyllene was dominant, while β-myrcene and limonene were among the major monoterpenes identified. 

Among the cannabis strains analyzed by Shapira, Berman, Futoran, Guberman, and Meiri [75], five chemotype groups were elucidated according the predominant terpenes: (i) β-myrcene, (ii) α- and β-pinene, (iii) β-caryophyllene and limonene, (iv) β-caryophyllene, and (v) terpinolene. In the sensory perception of the terpene profile differences among cannabis strains, two distinct descriptive clustering groups were nominated [71]. The first group included uniformly earthy, woody, and herbal, and the other group comprised the most frequent descriptors including citrus, lemon, sweet, and pungent. Table 2 shows the lists of cannabis strains available from the Dutch passion seed company (https://dutch-passion.com) classified by the chemotypes and descriptive ategories.

## 6. Separation of Cannabis Terpenes and Industrial Importance

In the past, identifying the terpene profile of the cannabis was for the purposes of improving canine training aids in illicit drug detection [76]. In the world of the cannabis industry, however, terpenes play a vital role in differentiating the flavor and aroma that are specific to the particular strains [56]. Some terpenes can enhance the effect of cannabinoids and synergize the feeling of relaxation, stress relief, energy boost, and maintaining focus along with their underlying pharmaceutical functions [54,79]. Thus, a growing number of industries have shown interest in adding either cannabis terpenes or botanically-derived terpenes to their CBD oils and edibles. The estimated growth in this sector should reach a 20 billion-market by 2024 [80,81]. The success in this sector might be challenged by a few restrains. First, the consumer believes that the functionality and safety are truly related to sources, perceived novelty, and most importantly, perceived benefits [82,83]. Additionally, the extraction of the full-spectrum oil consisting of a full mix of naturally occurring cannabis terpenes is almost impossible. The most cost-effective way is to selectively separate the terpenes and include them back into the final products [68,80]. 

Numerous terpene recovery techniques have been developed by solvent-based or solvent-less. Essential oils are usually hydro-distillated extracts from the trichomes of cannabis containing mostly terpenes or terpenoids. Although most of the constituents remain intact during distillation, a few monoterpenes may undergo chemical changes or are quite often lost due to the nature of the distillation process [26,56]. The other possible technique is steam distillation by passing dry steam through the inflorescences of the cannabis whereby the terpenes are volatilized, condensed, and collected [84,85]. Moreover, the microwave-assisted extraction (MAE) can enrich bioactive compounds. The MAE treatment using high irradiation power and relatively long extraction times significantly increased the content of CBD in the essential oil with considerably high yield when compared with the conventional hydro-distillation techniques [86]. In Canada, for instance, the commercial production of the extract is achieved by either solvent extraction such as butane or supercritical fluid (SFE) with the restriction on product purity of no solvent contaminants [87]. The later technique is known to give superior performance in terpene recovery [88]. SFE has recently become a much-preferred method for terpene recovery, largely because it allows using lower temperatures, leading to less deterioration of the thermally labile components and is free from organic solvents [88,89]. A supercritical fluid is the substance at a temperature and pressure above its critical points, with no boundary between the liquid and gas stage. At these points, the fluid is low in viscosity with high diffusion properties to dissolve chemical molecules from the plant matrix. Carbon dioxide (CO_2_) is generally used because it is nonflammable, relatively inexpensive, and non-toxic. Large amounts of terpene ingredients were recovered from this method (i.e., up to 50%, 20% and 10% of caryophyllene, humulene and limonene, respectively, can be recovered compared to the conventional methods) [64,89]. 

## 7. Conclusions

Recreational cannabis as a food ingredient has become more acceptable in a broader public context in which cannabis terpenes have gained high industrial attention in recent years. The terpene profiles not only embody the characteristics of cannabis genotypes, but their entourage effect with cannabinoids could enhance their medicinal functionality. This review highlights the importance of understanding cannabis terpene chemistry and provide descriptive profile categories of different cannabis commercial strains.

## Figures and Tables

**Table 1:** Differences between cannabis (marijuana) and hemp.

Characteristics	Cannabis (Marijuana)	Hemp
Genus	*Cannabis sativa* L.	*Cannabis sativa* L.
Sub variety	Indica	sativa
Utilized organs	leaves, flowers, stems and seeds containing trichomes	stem
Level of psychoactive THC	High (>1%/DW)	Low
Medicinal CBD	Can be high	Can be high
Leaf	Broad, darker leaf color	Thinner and greenish
Content of terpene (Rosin)	High (gluey)	Low

**Table 2:** Terpene profile category of different commercial cannabis stains.

Cannabis Family (commercial)*	**Stains (Commercial Names) **	**Seed Types**	**Level of Cannabinoid THC (Max = 5)**	**Chemotypic Catagories ^1^**					**Descriptive Sensorial Categories ^1^**	
				**(i) β-myrcene**	**(ii) α- and β-pinene**	**(iii) β-caryophyllene and Limonene**	**(iv) β-caryophyllene**	**(v) Terpinolene**	**(i) Earthy, Woody and Herbal**	**(ii) Citrus, Lemon, Sweet and Pungent**
**Afghani Kush**	Banana Blaze^®^	F	3		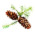					
	Auto Banana Blaze^®^	F, A	5					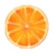		
	Master Kush	F	3		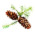					
	Night Queen^®^	F	4			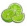				
**Blue family**	Auto Blue Berry^®^	F, A	3			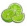				
	Auto Black Berry Kush^®^	F, A	4			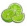				
	Blue Auto Mazar^®^	F, A	4		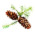		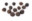			
**CBD rich**	CBD Charlotte’s Angel^®^	F	1	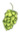		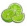				
	CBD Skunk Haze^®^	F	2		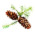		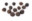			
**Classics**	C-vibez^®^	F	5	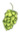						
	Mokum’s Tulip^®^	F	4		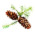					
	Auto Ultimate^®^	F, A	4	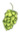	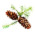					
	Think Fast^®^	F	3				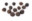			
	Auto Cinderella Jack^®^	F, A	5	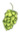	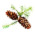					
	Outlaw Amnesia^®^	F	4	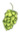	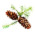					
	Auto Xtreme^®^	F, A	4		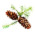		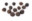			
	Auto White Widow^®^	F, A	4		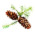		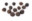			
**Dutch outdoor**	Frisian Dew^®^	F	2		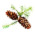		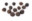			
	Purple N0. 1^®^	R, F	2		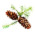		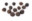			
	Auto Durban Poison^®^	F, A	2	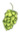			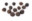			
**High altitude**	Snow Bud^®^	F	2	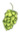		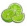				
**Orange family**	Passion Fruit^®^	F	4	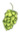	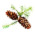					
**US special**	Sugar Bomb Punch^®^	F	5			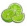	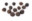			
	Kerosene Krash^®^	F	5			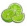				
	Meringue^®^	F	5			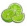				
	Hifi 4G^®^	F	4	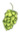		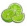				
	Auto lemon Kix^®^	F, A	5				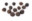	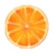		
	Bubba island Kush^®^	F	4			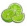				
	Auto Glueberry O.G.^®^	F, A	4	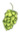			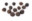			

^1^ The chemotype categories as described by Shapira, Berman, Futoran, Guberman, and Meiri [75] and the descriptive sensory group according to Gilbert and DiVerdi [71]. F = Feminized; A = Auto; R = Regular, * available at https://dutch-passion.com. The odor representatives; 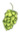 hop; 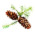 pine; 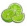 lime; 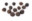 spice and 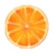 orange peel were according to Russo [28].

## References

[1] Cheng C., Zang G., Zhao L., Gao C., Tang Q., Chen J., Guo X., Peng D., Su J. (2016). A rapid shoot regeneration protocol from the cotyledons of hemp (*Cannabis sativa* L.). Ind. Crop. Prod..

[2] Weinberger A.H., Zhu J., Levin J., Barrington-Trimis J.L., Copeland J., Wyka K., Kim J.H., Goodwin R.D. (2020). Cannabis use among US adults with anxiety from 2008 to 2017: The role of state-level cannabis legalization. Drug Alcohol Depend..

[3] Rabiee R., Lundin A., Agardh E., Hensing G., Allebeck P., Danielsson A.-K. (2020). Cannabis use and the risk of anxiety and depression in women: A comparison of three Swedish cohorts. Drug Alcohol Depend..

[4] Lloyd S.L., Lopez-Quintero C., Striley C.W. (2020). Sex differences in driving under the influence of cannabis: The role of medical and recreational cannabis use. Addict. Behav..

[5] Turna J., Balodis I., Munn C., Van Ameringen M., Busse J., MacKillop J. (2020). Overlapping patterns of recreational and medical cannabis use in a large community sample of cannabis users. Compr. Psychiatry.

[6] Theparat C. New Rule Makes It Legal to Grow Hemp. https://www.bangkokpost.com/thailand/general/1845714/new-rule-makes-it-legal-to-grow-hemp.

[7] Koltai H., Namdar D. (2020). Cannabis Phytomolecule ‘Entourage’: From Domestication to Medical Use. Trends Plant Sci..

[8] Booth J.K., Bohlmann J. (2019). Terpenes in *Cannabis sativa*—From plant genome to humans. Plant Sci..

[9] Clarke R., Merlin M.D. (2019). History of Cannabis Use for Fiber. Cannabis.

[10] Fleming M., Clarke R. (1998). Physical evidence for the antiquity of *Cannabis sativa* L.. J. Int. Hemp Assoc..

[11] Li H.-L. (1973). An archaeological and historical account of cannabis in China. Econ. Bot..

[12] Li H.-L. (1974). The origin and use of cannabis in Eastern Asia linguistic-cultural implications. Econ. Bot..

[13] Hillig K.W. (2004). A chemotaxonomic analysis of terpenoid variation in Cannabis. Biochem. Syst. Ecol..

[14] Aluko R.E., Nadathur S.R., Wanasundara J.P.D., Scanlin L. (2017). Chapter 7—Hemp Seed (*Cannabis sativa* L.) Proteins: Composition, Structure, Enzymatic Modification, and Functional or Bioactive Properties. Sustainable Protein Sources.

[15] Abel E.L. (1980). Cannabis in the Ancient World. Marihuana.

[16] Bonini S.A., Premoli M., Tambaro S., Kumar A., Maccarinelli G., Memo M., Mastinu A. (2018). *Cannabis sativa*: A comprehensive ethnopharmacological review of a medicinal plant with a long history. J. Ethnopharmacol..

[17] Mediavilla V., Leupin M., Keller A. (2001). Influence of the growth stage of industrial hemp on the yield formation in relation to certain fibre quality traits. Ind. Crop. Prod..

[18] Cosentino S.L., Riggi E., Testa G., Scordia D., Copani V. (2013). Evaluation of European developed fibre hemp genotypes (*Cannabis sativa* L.) in semi-arid Mediterranean environment. Ind. Crop. Prod..

[19] Bewley-Taylor D., Blickman T., Jelsma M. (2014). The Rise and Decline of Cannabis Prohibition, the History of Cannabis in the UN Drug Control System and Options for Reform.

[20] Gaoni Y., Mechoulam R. (1964). Isolation, structure, and partial synthesis of an active constituent of Hashish. J. Am. Chem. Soc..

[21] Mudr P., Et S., Facultatis M., Facultatis M. (1964). Compounds. Acta Universitatis Palackianae Olomucensis-TOM.35.

[22] Adams R., Pease D.C., Cain C.K., Baker B.R., Clark J.H., Wolff H., Wearn R.B. (1940). Conversion of cannabidiol to a product with marihuana activity. A type reaction for synthesis of analogous substances. conversion of cannabidiol to cannabinol. J. Am. Chem. Soc..

[23] Devane W.A., Dysarz F.A., Johnson M.R., Melvin L.S., Howlett A.C. (1988). Determination and characterization of a cannabinoid receptor in rat brain. Mol. Pharmacol..

[24] Devane A.W., Hanus L., Breuer A., Pertwee R.G., Stevenson A.L., Griffin G., Gibson D., Mandelbaum A., Etinger A., Mechoulam R. (1992). Isolation and structure of a brain constituent that binds to the cannabinoid receptor. Science.

[25] Simonsen J.L., Todd A.R. (1942). 32. *Cannabis indica*. Part X. The essential oil from Egyptian hashish. J. Chem. Soc..

[26] Hanuš L.O., Hod Y. (2020). Terpenes/Terpenoids in Cannabis: Are They Important?. Med. Cannabis Cannabinoids.

[27] Ben-Shabat S., Fride E., Sheskin T., Tamiri T., Rhee M.-H., Vogel Z., Bisogno T., De Petrocellis L., Di Marzo V., Mechoulam R. (1998). An entourage effect: Inactive endogenous fatty acid glycerol esters enhance 2-arachidonoyl-glycerol cannabinoid activity. Eur. J. Pharmacol..

[28] Russo E.B. (2011). Taming THC: Potential cannabis synergy and phytocannabinoid-terpenoid entourage effects. Br. J. Pharmacol..

[29] Gallily R., Yekhtin Z., Hanuš L.O. (2018). The Anti-Inflammatory Properties of Terpenoids from Cannabis. Cannabis Cannabinoid Res..

[30] Baron E.P. (2018). Medicinal properties of cannabinoids, terpenes, and flavonoids in cannabis, and benefits in migraine, headache, and pain: An update on current evidence and cannabis science. Headache J. Head Face Pain.

[31] Lewis M.A., Russo E.B., Smith K.M. (2017). Pharmacological foundations of cannabis chemovars. Planta Med..

[32] Horne M.R.L., Kozłowski R.M., Mackiewicz-Talarczyk M. (2020). 5B-Bast fibres: Hemp cultivation and production. Handbook of Natural Fibres.

[33] Réquilé S., Le Duigou A., Bourmaud A., Baley C. (2018). Peeling experiments for hemp retting characterization targeting biocomposites. Ind. Crop. Prod..

[34] Anderson L.C. (1980). Leaf variation among cannabis species from a controlled garden. Bot. Mus. Leafl. Harv. Univ..

[35] Schultes R.E., Klein W.M., Plowman T., Lockwood T.E. (1975). 35. Cannabis: An Example of Taxonomic Neglect.

[36] Cai C., Yu W., Wang C., Liu L., Li F., Tan Z. (2019). Green extraction of cannabidiol from industrial hemp (*Cannabis sativa* L.) using deep eutectic solvents coupled with further enrichment and recovery by macroporous resin. J. Mol. Liq..

[37] Wiebelhaus N., Hamblin D., Kreitals N.M., Almirall J.R. (2016). Differentiation of marijuana headspace volatiles from other plants and hemp products using capillary microextraction of volatiles (CMV) coupled to gas-chromatography-mass spectrometry (GC-MS). Forensic Chem..

[38] Hammond C.T., Mahlberg P.G. (1977). Morphogenesis of capitate glandular hairs of *Cannabis sativa* (Cannabaceae). Am. J. Bot..

[39] Potter D. (2009). The Propagation, Characterisation and Optimisation of Cannabis sativa L. as a Phytopharmaceutical.

[40] Livingston S.J., Quilichini T.D., Booth J.K., Wong D.C.J., Rensing K.H., Laflamme-Yonkman J., Castellarin S.D., Bohlmann J., Page J.E., Samuels A.L. (2019). Cannabis glandular trichomes alter morphology and metabolite content during flower maturation. Plant J..

[41] Gupta A.K., Jain A., Roy P., Singh R. (2020). Pharmacological evaluation of *Cannabis indica* for their aphrodisiac potential. Int. J. Ayurvedic Med..

[42] Ahmed A., Shapiro D., Su J., Nelson L.P. (2020). Vaping Cannabis Butane Hash Oil Leads to Severe Acute Respiratory Distress Syndrome—A case of EVALI in a teenager with hypertrophic cardiomyopathy. J. Intensiv. Care Med..

[43] Stephens D., Patel J.K., Angelo D., Frunzi J. (2020). Cannabis butane hash oil dabbing induced lung injury mimicking atypical pneumonia. Cureus.

[44] Schwachtje J., Baldwin I.T. (2008). Why does herbivore attack reconfigure primary metabolism?. Plant Physiol..

[45] Sommano S., Visakh M.P., Iturriaga L.B., Ribotta P.D. (2013). Effect of Food Processing on Bioactive Compounds. Advances in Food Science and Nutrition.

[46] Sommano S.R., Caffin N., Kerven G. (2012). Screening for antioxidant activity, phenolic content, and flavonoids from Australian native food plants. Int. J. Food Prop..

[47] Sunanta P., Chung H.-H., Kunasakdakul K., Ruksiriwanich W., Jantrawut P., Hongsibsong S., Sommano S.R. (2020). Genomic relationship and physiochemical properties among raw materials used for Thai black garlic processing. Food Sci. Nutr..

[48] Wisetkomolmat J., Suppakittpaisarn P., Sommano S.R. (2019). Detergent plants of northern Thailand: Potential sources of natural saponins. Resources.

[49] Tangpao T., Chung H.-H., Sommano S.R. (2018). Aromatic profiles of essential oils from five commonly used Thai basils. Foods.

[50] Ross S.A., ElSohly M.A. (1996). The volatile oil composition of fresh and air-dried buds of *Cannabis sativa*. J. Nat. Prod..

[51] Turner C.E., ElSohly M.A., Boeren E.G. (1980). Constituents of *Cannabis sativa* L. XVII. A review of the natural constituents. J. Nat. Prod..

[52] Wanas A.S., Radwan M.M., Chandra S., Lata H., Mehmedic Z., Ali A., Baser K., Demirci B., ElSohly M.A. (2020). Chemical composition of volatile oils of fresh and air-dried buds of cannabis chemovars, their insecticidal and repellent activities. Nat. Prod. Commun..

[53] Rice S., Koziel J.A. (2015). Characterizing the smell of marijuana by odor impact of volatile compounds: An application of simultaneous chemical and sensory analysis. PLoS ONE.

[54] Ternelli M., Brighenti V., Anceschi L., Poto M., Bertelli D., Licata M., Pellati F. (2020). Innovative methods for the preparation of medical cannabis oils with a high content of both cannabinoids and terpenes. J. Pharm. Biomed. Anal..

[55] Wang C.-T., Ashworth K., Wiedinmyer C., Ortega J., Harley P.C., Rasool Q.Z., Vizuete W. (2020). Ambient measurements of monoterpenes near Cannabis cultivation facilities in Denver, Colorado. Atmos. Environ..

[56] Abdollahi M., Sefidkon F., Calagari M., Mousavi A., Mahomoodally M.F. (2020). Impact of four hemp (*Cannabis sativa* L.) varieties and stage of plant growth on yield and composition of essential oils. Ind. Crop. Prod..

[57] Sriwichai T., Junmahasathien T., Sookwong P., Potapohn N., Sommano S.R. (2019). Evaluation of the optimum harvesting maturity of makhwaen fruit for the perfumery industry. Agriculture.

[58] Booth J.K., Page J.E., Bohlmann J. (2017). Terpene synthases from *Cannabis sativa*. PLoS ONE.

[59] Nagegowda D.A., Gupta P. (2020). Advances in biosynthesis, regulation, and metabolic engineering of plant specialized terpenoids. Plant Sci..

[60] Chen F., Tholl D., Bohlmann J., Pichersky E. (2011). The family of terpene synthases in plants: A mid-size family of genes for specialized metabolism that is highly diversified throughout the kingdom. Plant J..

[61] Fellermeier M., Eisenreich W., Bacher A., Zenk M.H. (2001). Biosynthesis of cannabinoids Incorporation experiments with 13C-labeled glucoses. JBIC J. Biol. Inorg. Chem..

[62] Aliferis K.A., Bernard-Perron D. (2020). Cannabinomics: Application of metabolomics in cannabis (*Cannabis sativa* L.) research and development. Front. Plant Sci..

[63] Masoud A.N., Doorenbos N.J. (1973). Mississippi-Grown *Cannabis sativa* L. III: Cannabinoid and cannabinoid acid content. J. Pharm. Sci..

[64] Grijó D.R., Osorio I.A.V., Cardozo-Filho L. (2018). Supercritical extraction strategies using CO2 and ethanol to obtain cannabinoid compounds from Cannabis hybrid flowers. J. CO2 Util..

[65] Miyazawa M., Yamafuji C. (2005). Inhibition of acetylcholinesterase activity by bicyclic monoterpenoids. J. Agric. Food Chem..

[66] Gaggiotti S., Palmieri S., Pelle F.D., Sergi M., Cichelli A., Mascini M., Compagnone D. (2020). Piezoelectric peptide-hpDNA based electronic nose for the detection of terpenes; Evaluation of the aroma profile in different *Cannabis sativa* L. (hemp) samples. Sens. Actuators B Chem..

[67] Sriwichai T., Sookwong P., Siddiqui M.W., Sommano S.R. (2019). Aromatic profiling of *Zanthoxylum myriacanthum* (makwhaen) essential oils from dried fruits using different initial drying techniques. Ind. Crop. Prod..

[68] Maayah Z.H., Takahara S., Ferdaoussi M., Dyck J.R. (2020). The molecular mechanisms that underpin the biological benefits of full-spectrum cannabis extract in the treatment of neuropathic pain and inflammation. Biochim. Biophys. Acta (BBA) Mol. Basis Dis..

[69] Rao V.S.N., Menezes A.M.S., Viana G.S.B. (1990). Effect of myrcene on nociception in mice. J. Pharm. Pharmacol..

[70] Meschler J. (1999). Thujone exhibits low affinity for cannabinoid receptors but fails to evoke cannabimimetic responses. Pharmacol. Biochem. Behav..

[71] Gilbert A.N., DiVerdi J.A. (2018). Consumer perceptions of strain differences in Cannabis aroma. PLoS ONE.

[72] Brenneisen R., ElSohly M.A. (2007). Chemistry and Analysis of Phytocannabinoids and Other Cannabis Constituents. Marijuana and the Cannabinoids.

[73] Fischedick J.T., Hazekamp A., Erkelens T., Choi Y.H., Verpoorte R. (2010). Metabolic fingerprinting of *Cannabis sativa* L., cannabinoids and terpenoids for chemotaxonomic and drug standardization purposes. Phytochemistry.

[74] Brown A.K., Xia Z., Bulloch P., Idowu I., Francisco O., Stetefeld J., Stout J., Zimmer J., Marvin C., Letcher R.J. (2019). Validated quantitative cannabis profiling for Canadian regulatory compliance—Cannabinoids, aflatoxins, and terpenes. Anal. Chim. Acta.

[75] Shapira A., Berman P., Futoran K., Guberman O., Meiri D. (2019). Tandem Mass Spectrometric Quantification of 93 terpenoids in cannabis using static headspace injections. Anal. Chem..

[76] Rice S., Koziel J.A. (2015). The relationship between chemical concentration and odor activity value explains the inconsistency in making a comprehensive surrogate scent training tool representative of illicit drugs. Forensic Sci. Int..

[77] Kabir A., Holness H., Furton K.G., Almirall J.R. (2013). Recent advances in micro-sample preparation with forensic applications. TrAC Trends Anal. Chem..

[78] Calvi L., Pentimalli D., Panseri S., Giupponi L., Gelmini F., Beretta G., Vitali D., Bruno M., Zilio E., Pavlovic R. (2018). Comprehensive quality evaluation of medical *Cannabis sativa* L. inflorescence and macerated oils based on HS-SPME coupled to GC–MS and LC-HRMS (q-exactive orbitrap^®^) approach. J. Pharm. Biomed. Anal..

[79] Koltai H., Poulin P., Namdar D. (2019). Promoting cannabis products to pharmaceutical drugs. Eur. J. Pharm. Sci..

[80] Koby M. (2020). How Terpenes Could Revolutionize the Cannabis Industry as We Know It in Innovators.

[81] King J.W. (2019). The relationship between cannabis/hemp use in foods and processing methodology. Curr. Opin. Food Sci..

[82] Charlebois S., Somogyi S., Sterling B. (2018). Cannabis-infused food and Canadian consumers’ willingness to consider “recreational” cannabis as a food ingredient. Trends Food Sci. Technol..

[83] Khan R.S., Grigor J.V., Winger R., Win A. (2013). Functional food product development—Opportunities and challenges for food manufacturers. Trends Food Sci. Technol..

[84] Benelli G., Pavela R., Petrelli R., Cappellacci L., Santini G., Fiorini D., Sut S., Dall’Acqua S., Canale A., Maggi F. (2018). The essential oil from industrial hemp (*Cannabis sativa* L.) by-products as an effective tool for insect pest management in organic crops. Ind. Crop. Prod..

[85] Hanif M.A., Nawaz H., Naz S., Mukhtar R., Rashid N., Bhatti I.A., Saleem M. (2017). Raman spectroscopy for the characterization of different fractions of hemp essential oil extracted at 130 °C using steam distillation method. Spectrochim. Acta Part A Mol. Biomol. Spectrosc..

[86] Fiorini D., Scortichini S., Bonacucina G., Greco N.G., Mazzara E., Petrelli R., Torresi J., Maggi F., Cespi M. (2020). Cannabidiol-enriched hemp essential oil obtained by an optimized microwave-assisted extraction using a central composite design. Ind. Crop. Prod..

[87] Blake A., Nahtigal I. (2019). The evolving landscape of cannabis edibles. Curr. Opin. Food Sci..

[88] Baldino L., Scognamiglio M., Reverchon E. (2020). Supercritical fluid technologies applied to the extraction of compounds of industrial interest from *Cannabis sativa* L. and to their pharmaceutical formulations: A review. J. Supercrit. Fluids.

[89] Naz S., Hanif M.A., Bhatti H.N., Ansari T.M. (2017). Impact of supercritical fluid extraction and traditional distillation on the isolation of aromatic compounds from *Cannabis indica* and *Cannabis sativa*. J. Essent. Oil Bear. Plants.

